# Completed suicide and gender equality: Sex and age specific five-year data from Turkey

**DOI:** 10.1192/j.eurpsy.2021.2203

**Published:** 2021-08-13

**Authors:** İ.G. Yılmaz Karaman

**Affiliations:** Psychiatry Department, Eskişehir Osmangazi University, Faculty of Medicine, Eskişehir, Turkey

**Keywords:** completed suicide, Turkey, gender equity, gender equality

## Abstract

**Introduction:**

Suicide is a public health problem which has biopsychosocial aspects. These three compartments function differently for women and men in terms of biology and gender inequality.

**Objectives:**

This study aims to investigate completed suicide rates in Turkey for women and men seperately considering age ranges for each, and their relationship with gender equality.

**Methods:**

Sex and age specific data between 2015-2019 was derived from Turkish Statistical Institute. Utilizing Bağdatlı Kalkan’s study (2018) and Turkey’s Gender Equality Ratings (2019), 81 cities were seperated into two clusters (Table 1). Mann Whitney U and Independent Samples T Test were applied.

**Results:**

Young women’s (<30 years old) crude completed suicide rates were higher, when crude completed suicide rates for men over the age of 30 were fewer in the cities which equality index is low (Table 2). Regardless of age ranges, in better gender equality cluster, female suicide rates were fewer, male suicide rates were higher. The number of deaths by suicide in 1000 deaths didn’t differ for men, while the rate decreases for women in better gender equality cluster (Table 3).

**Figure:**
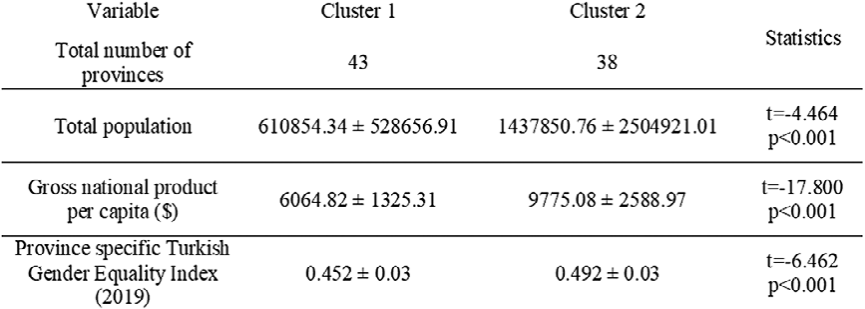

**Figure:**
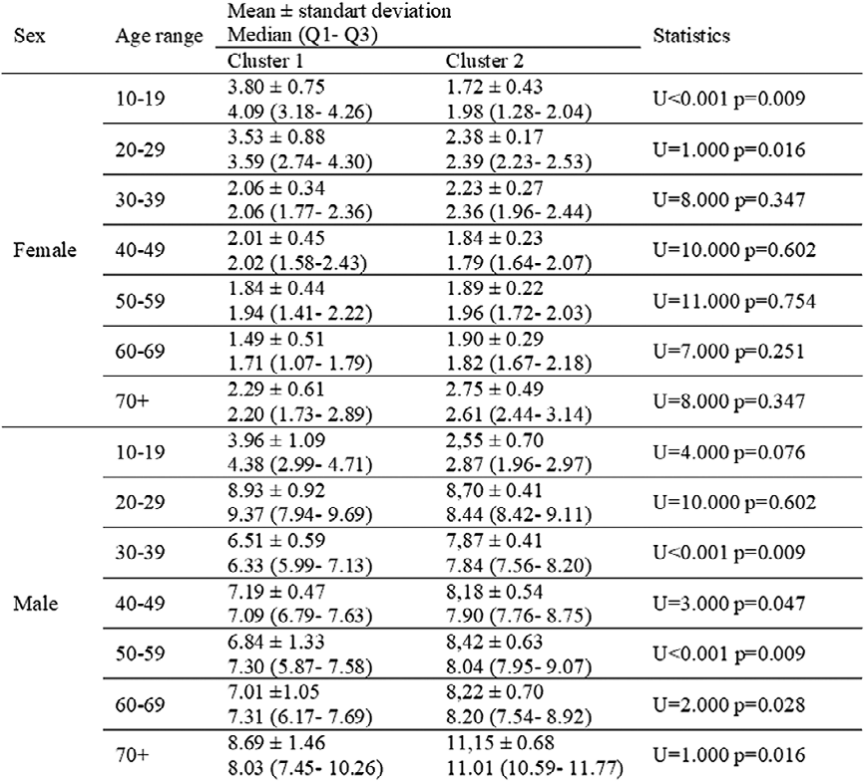

**Figure:**
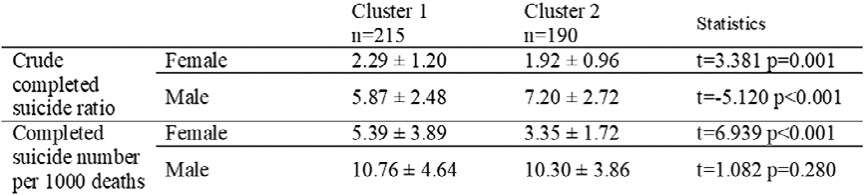

**Conclusions:**

Gender inequality may negatively effect young women’s mental health in more patriarchal cities in Turkey from the point of completed suicide.

**Disclosure:**

No significant relationships.

